# Wrinkled1 Accelerates Flowering and Regulates Lipid Homeostasis between Oil Accumulation and Membrane Lipid Anabolism in *Brassica napus*

**DOI:** 10.3389/fpls.2015.01015

**Published:** 2015-11-19

**Authors:** Qing Li, Jianhua Shao, Shaohua Tang, Qingwen Shen, Tiehu Wang, Wenling Chen, Yueyun Hong

**Affiliations:** National Key Laboratory of Crop Genetic Improvement, College of Life Science and Technology, Huazhong Agricultural UniversityWuhan, China

**Keywords:** Wrinkled1 (WRI1), oil accumulation, flowering, lipid homeostasis, transcriptional regulation, *Brassica napus*

## Abstract

Wrinkled1 (WRI1) belongs to the APETALA2 transcription factor family; it is unique to plants and is a central regulator of oil synthesis in *Arabidopsis*. The effects of WRI1 on comprehensive lipid metabolism and plant development were unknown, especially in crop plants. This study found that BnWRI1 in *Brassica napus* accelerated flowering and enhanced oil accumulation in both seeds and leaves without leading to a visible growth inhibition. BnWRI1 decreased storage carbohydrates and increased soluble sugars to facilitate the carbon flux to lipid anabolism. BnWRI1 is localized to the nucleus and directly binds to the AW-box at proximal upstream regions of genes involved in fatty acid (FA) synthesis and lipid assembly. The overexpression (OE) of Bn*WRI1* resulted in the up-regulation of genes involved in glycolysis, FA synthesis, lipid assembly, and flowering. Lipid profiling revealed increased galactolipids monogalactosyldiacylglycerol (MGDG), digalactosyldiacylglycerol (DGDG), and phosphatidylcholine (PC) in the leaves of OE plants, whereas it exhibited a reduced level of the galactolipids DGDG and MGDG and increased levels of PC, phosphatidylethanolamide, and oil [triacylglycerol (TAG)] in the siliques of OE plants during the early seed development stage. These results suggest that BnWRI1 is important for homeostasis among TAG, membrane lipids and sugars, and thus facilitates flowering and oil accumulation in *B. napus*.

## Introduction

Lipids not only serve as storage components of high-density energy, but they also function as essential components of cell membranes and regulators of various cellular processes during growth, development, and stress responses (Wang et al., 2006; Hong et al., 2008, 2009; Phillips et al., 2009; To et al., 2012). Fatty acid (FA) synthesis and lipid assembly involve multiple steps (Li-Beisson et al., 2010). The initial precursors of lipid biosynthesis include acetyl-CoA and glycerol-3-phosphate, which are initially derived from glycolysis and the Calvin–Benson cycle in plants (Kang and Rawsthorne, 1996; Alonso et al., 2007). The acetyl-CoA carboxylase (ACCase) complex is made of three subunits, namely biotin carboxyl carrier protein (BCCP), biotin carboxylase (BC), and carboxyltransferase (CT); this complex is encoded by separated genes in plants and catalyzes acetyl-CoA and CO_2_ to produce malonyl-CoA, the first committed step in *de novo* FA synthesis (Slabas and Fawcett, 1992; Ohlrogge and Browse, 1995; Ohlrogge and Jaworski, 1997; Voelker and Kinney, 2001; Sasaki and Nagano, 2004). Malonyl-CoA was then transferred to ACP protein by malonyl-CoA: ACP transferase (MAT) to initiate FA synthesis, and malonyl-CoA provides a two-carbon unit for acyl chain elongation as catalyzed by an FA synthase (FAS) complex (Ohlrogge and Browse, 1995). The synthesized FAs are either retained in the chloroplast for galactolipid synthesis or exported to the endoplasmic reticulum (ER) for membrane phospholipid and storage lipid [triacylglycerol (TAG)] assembly. The final step of TAG assembly as catalyzed by DAG acyltransferase (DGAT), which occurs in the Kennedy pathway, is also regarded as a critical reaction for oil accumulation (Slabas and Fawcett, 1992; Ohlrogge and Browse, 1995; Zou et al., 1999; Voelker and Kinney, 2001). The loss of DGAT1 resulted in reduced seed oil content, whereas *DGAT1* overexpression (OE) enhanced the seed oil content in *Arabidopsis* (Zou et al., 1999; Jako et al., 2001). The OE of maize high-oil *DGAT1-2* also promoted oil accumulation in maize seeds (Zheng et al., 2008). Alternatively, phosphatidylcholine (PC) also provides an acyl chain toward DAG for TAG synthesis, as catalyzed by phospholipid:diacylglycerol acyltransferase (PDAT; Dahlqvist et al., 2000; Tarczynski and Shen, 2008; Zhang et al., 2009).

Given the complicated networks that make up the lipid anabolic process, it would be more efficient to boost oil accumulation by enhancing multiple routes in a coordinated fashion including carbon partitioning, FA synthesis, and lipid assembly. Therefore, the identification of the key enzymes or master regulators involved in multiple steps simultaneously becomes an attractive approach for improving oil production (Ohlrogge and Jaworski, 1997; Ruuska et al., 2002; Cahoon et al., 2007; Mu et al., 2008). Transcriptomic profiling revealed that the genes encoding enzymes involved in FA synthesis is co-regulated to the rate of acyl chain synthesis, suggesting that transcriptional regulation plays an important role in the lipid biosynthesis process (Ruuska et al., 2002; Baud and Lepiniec, 2009; Barthole et al., 2012). Recent studies have identified several transcription factors that are capable of governing multiple oil accumulation steps (Cernac and Benning, 2004; Shen et al., 2010; To et al., 2012). Wrinkled1 (WRI1) belongs to the APETALA2 (AP2)-ethylene-responsive element binding protein family of transcription factors, and it acts as a central regulator in seed oil accumulation by modulating numerous genes simultaneously during late glycolysis and FA biosynthesis. A deficiency mutant of *Arabidopsis WRI1* (At*WRI1*) leads to wrinkled seeds with 80% less seed oil content in *Arabidopsis* (Focks and Benning, 1998; Cernac and Benning, 2004). The loss of AtWRI1 also leads to impaired seed germination and seedling establishment, whereas At*WRI1* OE enhances oil accumulation in *Arabidopsis*, which is accompanied by aberrant seedling development (Stone et al., 2001; Kwong et al., 2003; Cernac and Benning, 2004; Cernac et al., 2006; Baud et al., 2007). AtWRI1 binds to the AW-box consensus [CnTnG](n)_7_[CG] in the proximal promoter of target genes that are involved in the glycolysis and FA synthesis of *Arabidopsis* (Maeo et al., 2009; To et al., 2012).

The biological significance of WRI1 has been extensively studied in relation to oil accumulation in *Arabidopsis*. The role of WRI1 in comprehensive lipid regulation in other plant species, particularly in crop plants, remains to be elucidated. A recent study showed that the WRI1 homolog from maize is able to compensate for the impaired oil accumulation and seedling establishment of the At*wri1* mutant in *Arabidopsis* (Cernac et al., 2006; Pouvreau et al., 2011). The OE of Zm*WRI1* in maize increased the levels of FAs and some amino acid residues (Pouvreau et al., 2011), suggesting that the role of WRI1 in oil accumulation is highly conserved between monocot and dicot plants. *Arabidopsis* that overexpresses At*WRI1* exhibits undesirable agronomic traits, with retarded growth and reduced biomass (Lotan et al., 1998; Stone et al., 2001; Cernac and Benning, 2004; Wang et al., 2007; Mu et al., 2008), whereas Zm*WRI1* OE in maize promotes oil accumulation without visible side effects on growth and development (Shen et al., 2010). The results suggest that the role of WRI1 in oil synthesis is conserved but distinguishable in different plant species. The WRI1 in different plant species may exhibit a unique role in addition to its effect on oil accumulation. Furthermore, most WRI1 studies have been focused on oil accumulation, and the effects of WRI1 on membrane phospholipids and galactolipids have remained unknown. In the present study, we characterized BnWRI1 (BnaA09g34250D) from *Brassica napus* (*B. napus*). Bn*WRI1* OE in *B. napus* resulted in enhanced lipid anabolism by binding to the *cis*-element CnTnG (n)_7_CG in the promoter regions of genes involved in FA synthesis and lipid assembly to up-regulate these target genes. BnWRI1 promotes oil accumulation and thylakoid membrane monogalactosyldiacylglycerol (MGDG), digalactosyldiacylglycerol (DGDG), and PC biosynthesis to regulate homeostasis among membrane lipids, oils, and carbohydrates. Therefore, Bn*WRI1* OE facilitates flowering, reproduction, and oil production without visible side effects on the growth of *B. napus*.

## Materials and Methods

### Plant Materials and Growth Conditions

The Westar cultivar of canola (*B. napus* L.) was used in this study. Seeds were germinated in either Murashige and Skoog (MS) plates or soil in pots. Three-week-old seedlings were then transferred to pots containing soil. The plants were raised in a growth room under 16 h light (25°C)/8 h dark (20°C), a photosynthetic photon flux density of 200–300 mmol m^-2^ s^-1^, and 60% relative humidity, or natural conditions during winter-spring seasons in Wuhan, China. For the field growth test, 3-week-old seedlings were transferred to the field at suitable spacing (33 cm × 50 cm) that were arranged in a one-way randomized block design with 30 plants/lines per block, and three replications.

### Gene Cloning, Vector Construction, and *B. napus* Plant Transformation

To obtain the full-length Bn*WRI1* cDNA, total RNA was extracted from the leaves of 4-week-old *B. napus* plants, and it was subjected to reverse transcription to obtain first strand cDNA according to the manufacturer’s instructions (TransGene Biotech, Beijing, China). The full-length Bn*WRI1* cDNA was amplified by PCR with primers Bn*WRI1*F 5′-GGATCCATGAAGAGACCCTTAACCACT-3′ and Bn*WRI1*R 5′-GAGCTCTCAGACAGAATAGTTCCAAGAA-3′, and then it was ligated into binary vector pBI121, which had been digested by *Sac*I and *BamH*I. The resulting construct was transformed into *B. napus* by *Agrobacterium* GV3101 mediation with hypocotyls used as explants for regeneration. The transgenic shoots were first selected on kanamycin (50 μg/ml), and then the kanamycin resistant shoots were transferred to MS medium containing 1-naphthaleneacetic acid for rooting. The transgenic plants were further confirmed by PCR with a pBI121 vector and Bn*WRI1* sequence specific primers (Supplemental Table S1).

### Subcellular Localization

The full-length cDNA of Bn*WRI1* was ligated into pCAMBIA1301 vector that had been digested by the restriction enzymes *Sac*I and *BamH*I. The construct containing Bn*WRI1*-GFP was introduced into *Agrobacterium* GV3101 and infiltrated into tobacco leaves for 24 h to obtain transient protein expression under the control of the 35S promoter. Subcellular localization was visualized under a confocal laser scanning microscope (Leica, Biberach, Germany) with the exciter filter HFT488 and the transmitting optical filter BP505–530 to observe the green fluorescence. The nuclei were labeled with 4′,6-diamidine-2-phenylindole dihydrochloride (DAPI) staining.

### RNA Extraction and Quantitative Real-time PCR

Total RNA was extracted from various tissues at different stages using TransZol reagent (TransGen Biotech, Beijing, China), and it was then treated with RNase-free DNaseI (NEW ENGLAND Biolabs, Ipswitch, MA, USA) to remove any contaminating DNA. The resulting RNA was used for first strand synthesis by reverse transcriptase with an oligo-d (T) 18 primer (TransGen Biotech, Beijing, China) to obtain cDNA according the manufacturer’s protocol. Quantitative real-time PCR was performed with SYBR Green PCR Master Mix (TransGen Biotech, Beijing, China) on a single-color Real-time PCR Detection System (Bio-Rad, Hercules, CA, USA). A Bn*Actin* gene was used as the standard control. The quantitative real-time PCR conditions were as follows: 95°C for 1 min; 40 cycles of 95°C for 30 s, 55°C for 30 s, 72°C for 30 s; and 72°C for 10 min for the final extension. The primers used for real-time PCR are listed in Supplemental Table S2.

### Lipid Extraction and Analyses

Lipids were extracted from the leaves, developing siliques, and mature seeds. The lipids were separated on a thin layer chromatography (TLC) plate with developing solvent consisting of petroleum ether, ethyl ether, and acetic acid (80:20:1, v/v). The separated lipids were visualized with iodine vapor and the spots were scraped for measurement by GC analysis (Agilent 7890A, Santa Clara, CA, USA) after a methyl ester reaction with methanol and toluene containing 5% H_2_SO_4_ at 80°C for 3–4 h. To measure the seed oil contents, oil was extracted from the seeds and tested by GC analysis after the methyl ester reaction as detailed above. The GC running conditions were as follows: the injection port temperature was 180°C, and the oven temperature was set at 180°C for 2 min and was increased by 10°C/min up to 220°C for 5 min. The temperature of the flame ionization detector was 280°C with flow rates of 30, 300, and 25 ml/min for hydrogen, air, and helium, respectively.

### Electrophoretic Mobility-shift Assay

The full-length cDNA of Bn *WRI1* was amplified by using the forward primer 5′-CCCGGGTATGAAGAGACCCTTAACCAC-3′ coupled with the reverse primer 5′-GGATCCCGACAGAATAGTTCCAAGAA-3′, and then it was ligated to pET28a vectors that were digested by *BamH*I and *Sac*I. This construct was transformed into *Escherichia coli* strain Rosetta (DE3), and the BnWRI1 protein was expressed by induction with 0.6 mM isopropyl-β-D-thiogalactopyranoside (IPTG) while the strain was grown in Luria Bertani (LB) medium overnight at 20°C. The cells were harvested and lysed by sonication in a buffer (300 mM NaCl, 20 mM Tris-HCl, pH 8.0, 10 mM imidazole, 5% glycerol, and 50 mM NaH_2_PO_4_). The cell lysate was centrifuged at 12,000 r/min for 20 min. The supernatant was incubated with Ni-NTA resin (Shanghai Sangon, http://www.sangon.com) for 3 h at 4°C. The BnWRI1 protein was eluted from the resin after three washes with wash buffer (50 mM NaH_2_PO_4_, 300 mM NaCl, and 20 mM imidazole, pH 8). Protein from *E. coli* cells containing only the pET28a vector was used as a negative control. The DNA sequences that were 300 and 250 bp upstream from the start codon of *KASI* and *GPAT9*, respectively, were amplified from *Arabidopsis* with the *KASI* forward primer 5′-GAATTCTGTTGAGTTACGAATTGGAG-3′, coupled with the *KASI* reverse primer 5′-GAGCTCATTGAGAGAGGTATTGAGAG-3′, and the *GPAT9* forward primer 5′-GAATTCACATAATATGTCCAAGATCATT-3′ coupled with the *GPAT9* reverse primer 5′-GAGCTCCTATTATACTTATACCACAT-3′. The substitutive nucleotide (C→T, T→C, G→A) mutant at the AW-box [CnTnG](n)_7_[CG] was amplified by using a similar approach. The amplified DNA fragments containing native or mutant AW-box were incubated with purified BnWRI1 protein in binding buffer (20 mM Tris-HCl, pH 8.0, 250 mM NaCl, 2 mM MgCl_2_, 1% glycerol, 1 mg/ml BSA, 1 mM DTT) for 1 h at 4°C. The resulting mixture was separated on native PAGE (6%) by electrophoresis and was visualized under UV light. The binding activity of BnWRI1 to the AW-box was also determined with biotin labeled DNA probes using a chemiluminescent electrophoretic mobility-shift assays (EMSA) kit (Beyotime, China) according to the manufacturer’s instructions.

### Measurements of Protein, Starch, and Soluble Sugar

Proteins were extracted from leaves and seeds by homogenizing in buffer containing 50 mM Tris-HCl, pH 8.0, 250 mM NaCl, 1 mM EDTA, and 1% (w/v) SDS, and incubating the mixture for 2 h at 25°C. The homogenate was centrifuged at 16,000 *g* for 10 min, the supernatant was diluted 200 times and the protein concentration was measured by Lowry D protein assay (Bio-Rad). Soluble sugars were measured using phenol-sulfuric acid method (DuBois et al., 1956; Chow and Landhausser, 2004). In brief, leaf samples (1 g fresh weight) were homogenized with deionized water and filtered. The extract (50 μl) was mixed with 450 μl of sulfuric acid containing anthrone (2 mg/ml) at 95°C, and then the absorbance at 625 nm was monitored by spectrometer (Infinite M200 PRD, Untersbergstr, Austria). For starch extraction, the remaining sediment was suspended in a solution containing 0.2 N KOH and incubated at 95°C for 1 h, followed by the addition of 1 N acetic acid and incubation for 15 min. After centrifugation at 16,000 *g* for 5 min, the starch in the supernatant was measured by using a method similar to that of soluble sugars.

## Results

### Expression Pattern and the Effect of BnWRI1 on Flowering in *B. napus*

**FIGURE 1:**
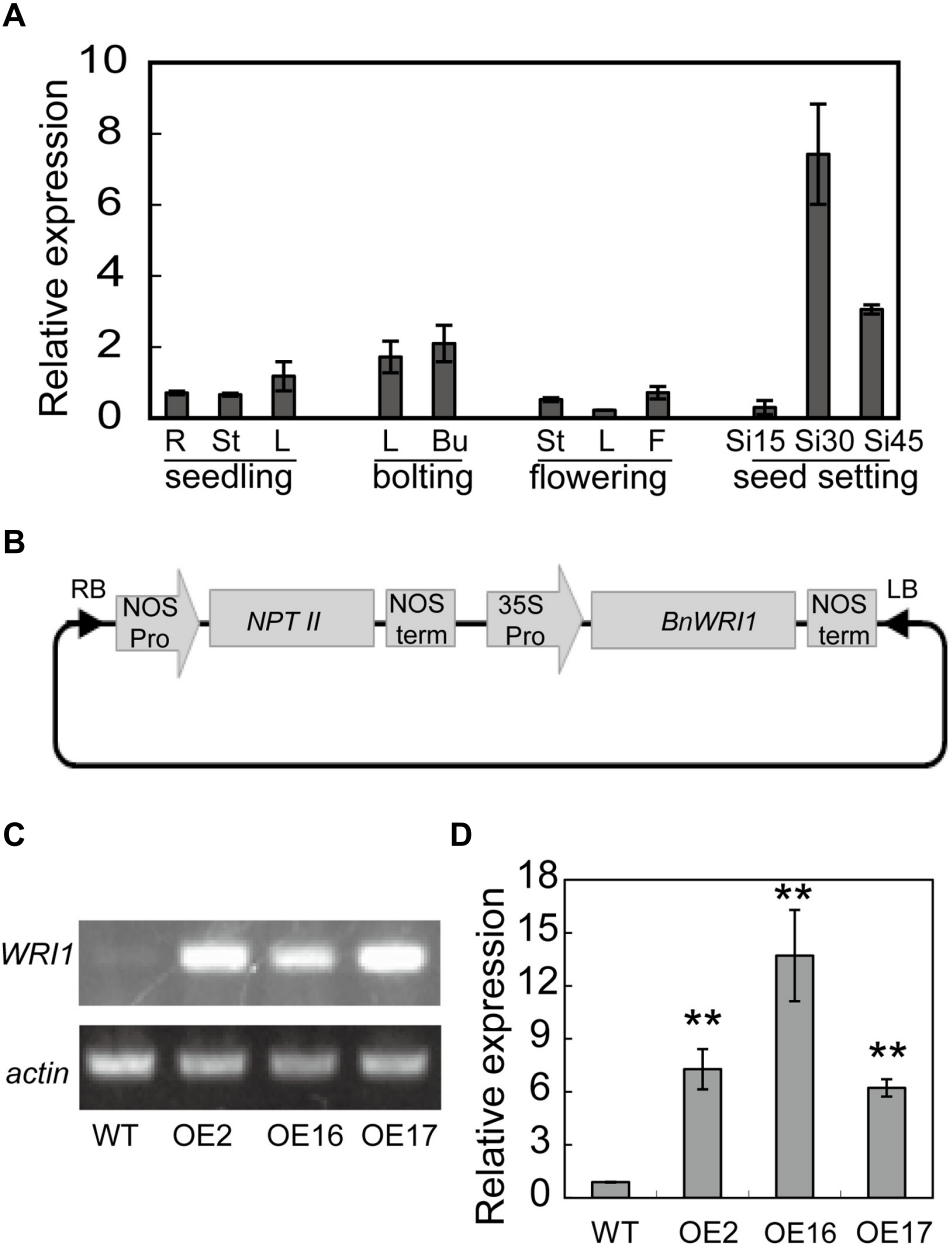
**Bn*WRI1* expression pattern and overexpression (OE) of Bn*WRI1* in *Brassica napus*. (A)** Transcript level of Bn*WRI1* in different *B. napus* tissues at seedling, bolting, flowering and seed developing stages. The expression level was quantified by real-time PCR normalized to the expression of Bn*Actin*. Values are mean ± SD (*n* = 3). R, root; St, stem; L, leaf; Bu, flower bud; F, flower; and Si15, Si30, and Si45, siliques at 15, 30, and 45 days, respectively, after anthesis. **(B)** The construct containing Bn*WRI1* in the binary vector pBI121. **(C,D)** The transcript level of Bn*WRI1* in Bn*WRI1-*OE plants according to semi-quantitative RT-PCR **(C)** and quantitative real-time PCR **(D)**. Total RNA was extracted from the leaves of 6-week-old plants, and the expression level was detected by using Bn*WRI1*-specific primers. The expression levels were normalized to Bn*Actin*. OE2, OE16, and OE16 represent Bn*WRI1*-OE lines. Values are mean ± SD (*n* = 3). ^∗∗^Indicates significant difference at *P* < 0.01 compared with the WT based on Student’s *t-*test.

To investigate the temporal and spatial distribution of Bn*WRI1* mRNA in *B. napus*, total RNA was extracted from various tissues at different stages and used for analysis by quantitative real-time PCR. During the seedling and bolting stages, the Bn*WRI1* transcript level was higher in leaves and flower buds than it was in roots and stems (**Figure 1A**). During the flowering stage, the Bn*WRI1* expression was higher in flowers than in leaves and stems. The transcript level was rapidly up-regulated in siliques and was highest at 30 days after anthesis (**Figure 1A**).

**FIGURE 2:**
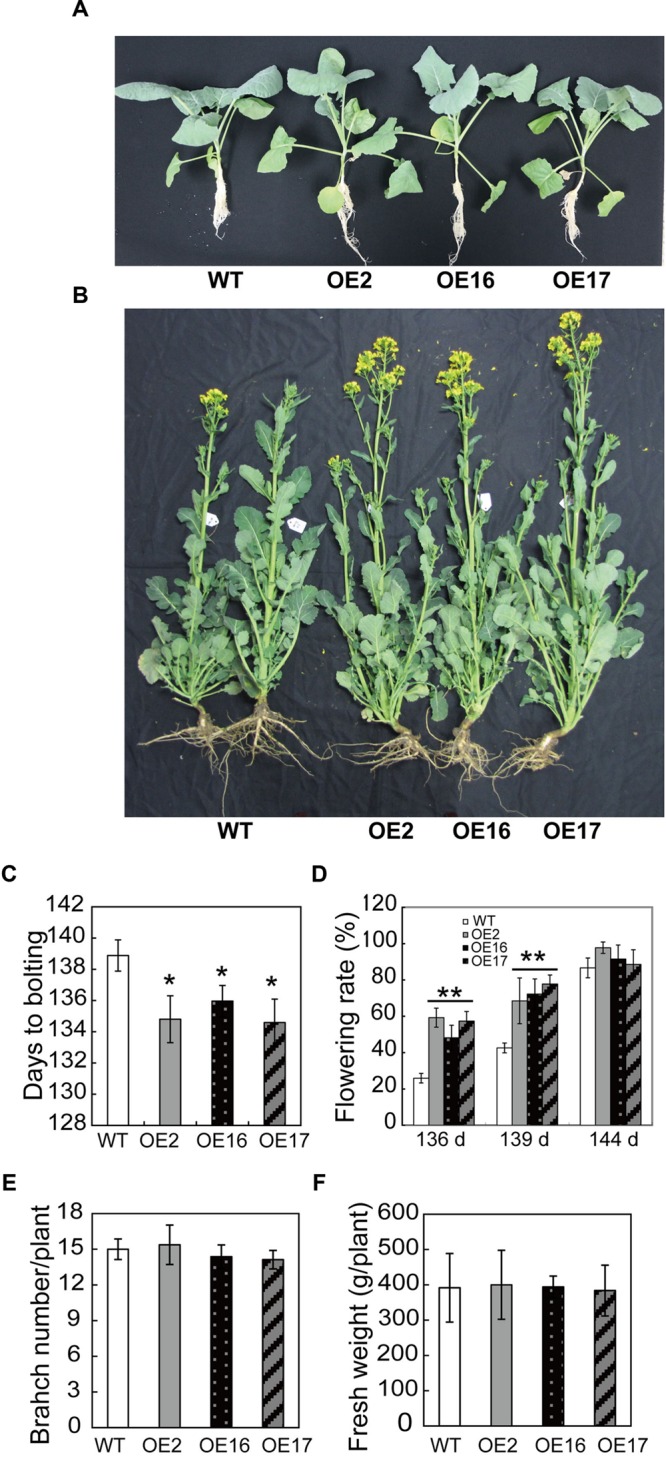
**Accelerated flowering in Bn*WRI1*-OE plants. (A)** Bn*WRI1-*OE and WT plants at the vegetative growth stage. The pictures were taken of plants that were grown in pots at 30 days after germination. **(B)** Accelerated flowering in Bn*WRI1-*OE *plants*. The picture was taken of 145-day-old plants grown in the field. **(C)** Days to bolting based on a flowering rate of 50% (*n* = 20, *r* = 3). **(D)** The flowering rate of OE and WT plants grown under the same conditions. Values are mean ± SD (*n* = 20, *r* = 3). **(E)** Inflorescent branch number of Bn*WRI1-*OE and WT. The data were collected from mature plants (175-day-old) grown in the field (*n* = 12, *r* = 3). **(F)** The fresh weights of aerial part from 175-day-old plants grown in the field. Values are mean ± SD (*n* = 12, *r* = 3). ^∗^,^∗∗^Indicate significant differences at *P* < 0.05 and *P* < 0.01, respectively, compared with the WT based on Student’s *t-*test.

To explore the biological function of BnWRI1 in *B. napus*, full-length Bn*WRI1* cDNA was cloned by reverse transcription PCR by using mRNA that was extracted from leaves as a template, and the cDNA was ligated into binary vector pBI121. The resulting construct containing Bn*WRI1* was transformed into *B. napus* under the control of the 35S promoter (**Figure 1B**). More than 30 independent transgenic lines were obtained, and the Bn*WRI1* transcript level in transgenic plants was much higher than that of the wild-type (WT; **Figures 1C,D**). Three representative, independent Bn*WRI1* OE lines, OE2, OE16, and OE17, were selected randomly from 30 transgenic lines, and they were used for further characterization. These plants were grown under natural conditions either in the field or in pots, and no visual growth change was observed between OE and WT plants, which showed a similar leaf size, leaf number, and growth rate during the vegetative growth stage (**Figure 2A**). However, BnWRI1 accelerated flowering; OE plants bolted and flowered 4 to 6 days earlier than WT plants (**Figures 2B,C**). At 136 days after germination, 60% of the OE plants were flowering, whereas only 23% of the WT plants were flowering (**Figure 2D**). The earlier flowering in OE plants did not cause changes in the total number of inflorescent branches and biomass at the mature stage compared with the results for the WT plants (**Figures 2E,F**).

### Overexpression of Bn*WRI1* Enhances Oil Accumulation in Seeds and Leaves without Undesirable Agronomic Traits

**FIGURE 3:**
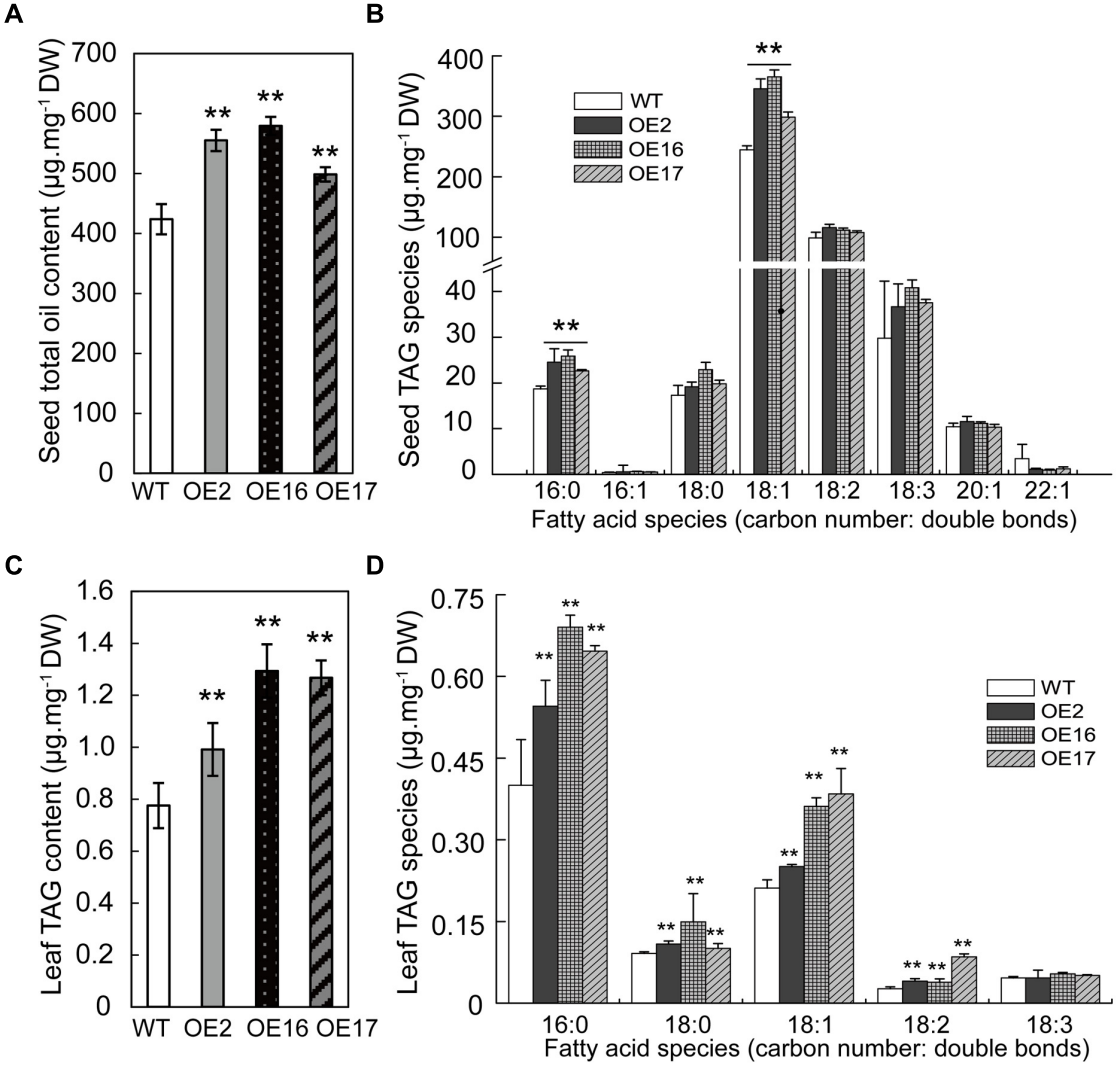
**Enhanced seed oil accumulation in Bn*WRI1-*OE plants.** Total TAG content **(A)** and fatty acid (FA) composition **(B)** in the seeds of Bn*WRI1*-OE and WT. Total TAG content **(C)** and FA composition **(D)** in the leaves of Bn*WRI1*-OE and WT. Lipids were extracted from the leaves of 8-week-old plants and were separated by using a thin layer chromatography (TLC) plate. TAG spots were scraped and extracted for methylation and GC measurement. Values are mean ± SD (*n* = 4); ^∗∗^*P* < 0.01.

To investigate the role of BnWRI1 in oil (TAG) synthesis, the oil content was measured in both the seeds and leaves of OE and WT plants. The oil content of Bn*WRI1*-OE seeds was significantly higher than that of the WT, and it was increased by 31, 38, and 18% in OE2, OE16, and OE17, respectively (**Figure 3A**). FA profiling revealed that oleic acid (18:1) contributed mostly to oil accumulation in OE plants. Other FA species such as 16:0 and 18:2 also increased in OE seeds (**Figure 3B**). The OE of Bn*WRI1* also increased the oil contents of vegetative tissues. The oils in the leaves were primarily composed of 16:0, 18:1, and 18:0 FA species, which account for ∼90% of the total FA species. The total TAG contents of the leaves from OE2, OE16, and OE17 increased by 28, 67, and 63%, respectively, in comparison with the WT plants (**Figure 3C**). The enhanced TAG in the OE leaves resulted from the increase of 16:0, 18:0, 18:1, and 18:2 FA species (**Figure 3D**). The relative increase in leaf oil content from Bn*WRI1* OE was greater than that of the seed oil content.

### The Effect of BnWRI1 on Total Lipids, Sugar, and Protein Accumulation

**FIGURE 4:**
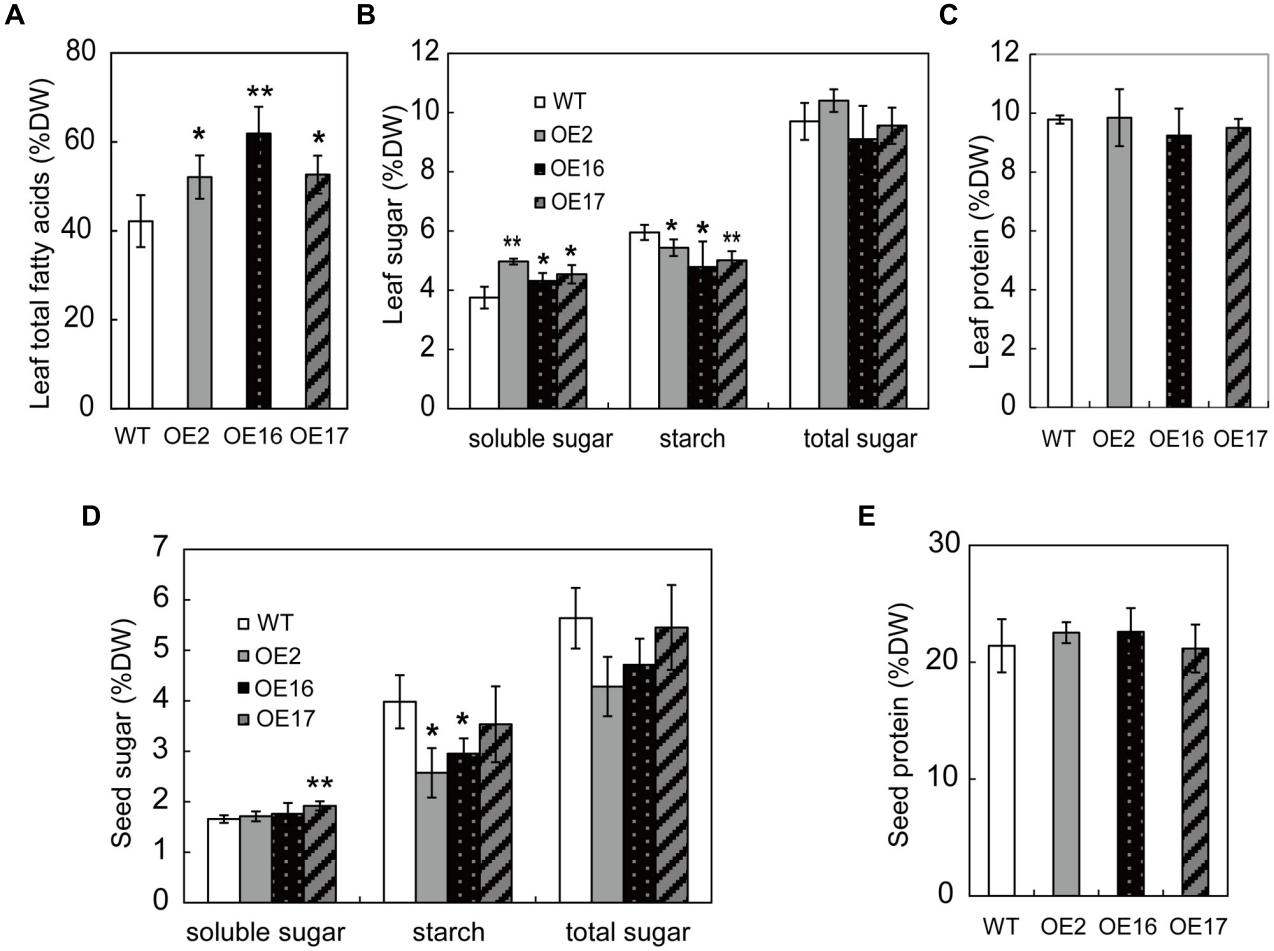
**The effect of BnWRI1 on partitioning among lipids, sugars, and proteins in *B. napus*. (A)** Total FA contents in leaves from Bn*WRI1-*OE and WT plants. **(B,C)** The soluble sugars, starch, total sugars, and proteins in the leaves of 2-month-old plants. **(D,E)** The soluble sugar, starch, total sugar, and protein contents in mature seeds. Values are mean ± SD (*n* = 3). DW, dry weight; ^∗^*P* < 0.05, ^∗∗^*P* < 0.01.

Carbohydrates are photosynthesized in green tissues, predominantly in leaves, which are the major source tissue, providing precursors for lipid and protein synthesis. Carbon partitioning among these metabolites is a major factor that influences lipid accumulation. To determine whether enhanced oil accumulation resulted from alterations in carbon partitioning, the contents of the total lipids, soluble sugars, starch, and proteins were measured in the leaves of 3-month-old plants at the flowering stage. The total lipid content of OE leaves was significantly higher than that of WT, and it was increased by 24, 47, and 25% for OE2, OE16, and OE17, respectively (**Figure 4A**). Bn*WRI1*-OE leaves exhibited increased soluble sugars with reduced starch contents compared with WT plants (**Figures 4B,C**). However, the total sugars and total protein content in OE leaves were not substantially different from those of WT plants (**Figures 4B,C**). In comparison with those in the leaves, the contents of soluble sugars, starch, total sugars, and proteins in mature seeds were less altered between the OE and WT (**Figures 4D,E**). The starch contents of OE2 and OE16 seeds were lower than that of the WT, whereas the soluble sugar, total sugar, and protein contents in OE seeds were not significantly different from that of the WT (**Figures 4D,E**). These results indicate that enhancing the lipid content by overexpressing Bn*WRI1* without reducing sugars and proteins, but enhanced leaf sugar moves from storage starch to soluble sugars for lipid accumulation.

### BnWRI1 Binds to the Proximal Upstream Regions of Genes Involved in Lipid Anabolism

**FIGURE 5:**
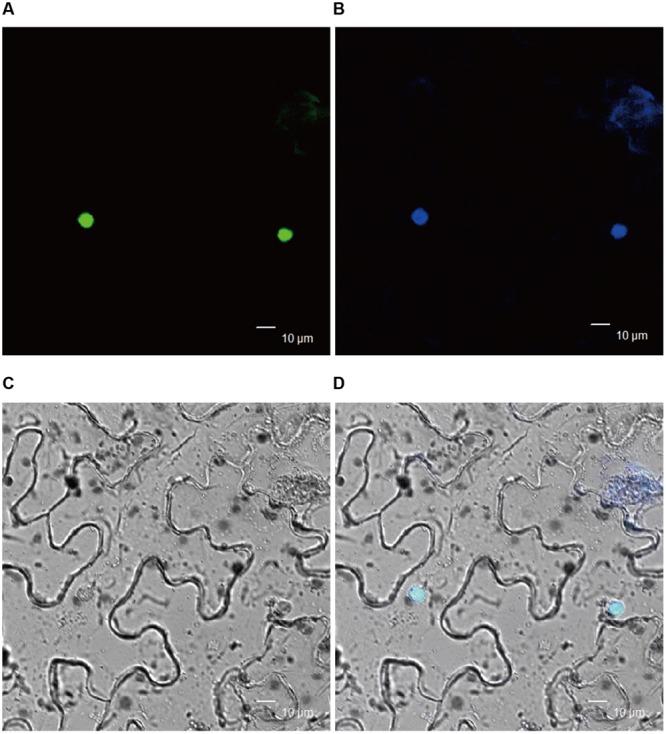
**Nuclear localization of BnWRI1. (A)** Green fluorescence of BnWRI1-GFP. **(B)** Nucleus stained with DAPI. **(C)** Bright image. **(D)** Merged image of the same cells observed in **(A)**. BnWRI1-GFP was transiently expressed in *Nicotiana benthamiana* epidermal cells and visualized under by confocal laser scanning microscopy. The nuclear localization of BnWRI1 was indicated by green fluorescence that was overlaid with the nucleus indicated by DAPI staining.

**FIGURE 6:**
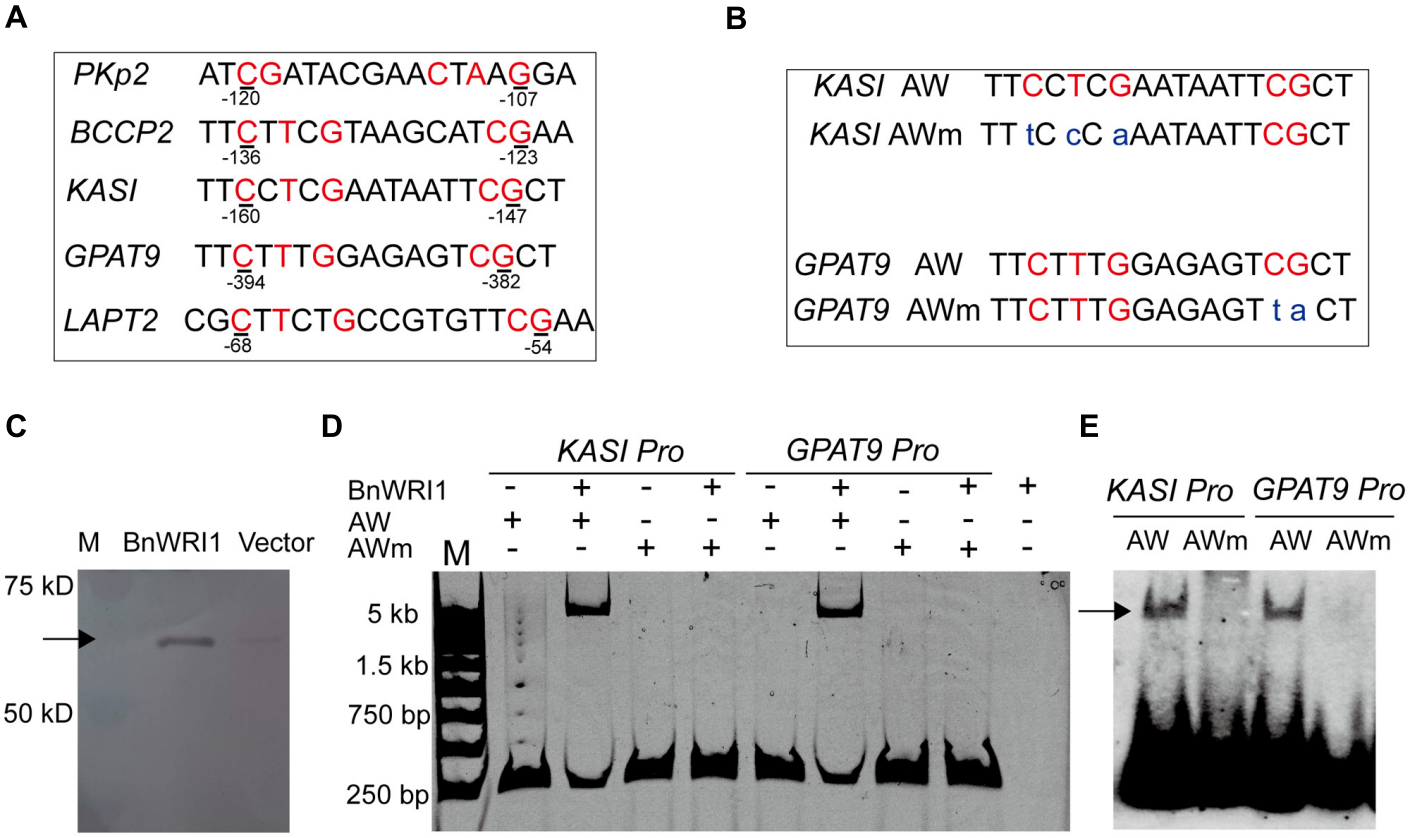
**BnWRI1 binding to proximal upstream regions of genes involved in lipid anabolism. (A)** The AW-box [CnTnG](n)_7_[CG] in the proximal upstream regions of genes involved in glycolysis, FA synthesis, and lipid assembly. **(B)** Consensus [CnTnG](n)_7_[CG] of the AW-box and its mutant AW-box [TnCnA](n)_7_[CG] of the *KASI* promoter or [CnTnG](n)_7_ [TA] of *GPAT9* promoter. **(C)** Recombinant BnWRI1 protein (50 kD) expressed in *E. coli* cells. **(D)** BnWRI1 specifically binds to the promoter region of *KASI* and *GPAT9* containing the AW-box as assayed by EMSA. The binding was abolished when the conserved sequence was mutated into the [TnCnA](n)_7_[CG] of the *KASI* promoter or the [CnTnG](n)_7_[TA] of the *GPAT9* promoter. The purified BnWRI1 protein (0.4 μg) was incubated with the target DNA fragment for 1 h. The resulting complex was visualized under UV light. The lanes from left to right: M, DNA ladder; *KASI* promoter only; BnWRI1 (0.4 μg) + *KASI* promoter; *KASI* mutant promoter only; BnWRI1 (0.4 μg) + *KASI* mutant promoter; *GPAT9* promoter only; BnWRI1 (0.4 μg) + *GPAT9* promoter; *GPAT9* mutant promoter only; BnWRI1 (0.4 μg) + *GPAT9* mutant promoter; BnWRI1 only. **(E)** The binding activity of BnWRI1 to the AW-box or its mutant AW-box (mAW) in the promoter regions of *KASI* and *GPAT9* by EMSA using the DNA probes labeled with biotin. The shifted band is indicated by the arrow.

To get insight into the molecular mechanism of BnWRI1 in lipid metabolism and carbon partitioning, the subcellular localization of BnWRI1 and its putative target genes involved in lipid metabolism were investigated. The full-length cDNA of Bn*WRI1* was fused with GFP at the C-terminus and then transiently expressed in the epidermal cells of tobacco leaves by *Agrobacterium* infiltration. Green fluorescent BnWRI1-GFP was visualized by confocal laser scanning microscopy (**Figure 5A**), and the resulting image was overlaid with a nucleus marked by 4′,6-diamidine-2-phenylindole dihydrochloride (DAPI) staining, confirming that the introduced BnWRI1 is localized to the nucleus (**Figures 5B–D**). The proximal upstream regions of *Arabidopsis* genes such as pyruvate kinase (*PKp), BCCP2, KASI, LPAT2*, and *GPAT9* that are involved in glycolysis, FA synthesis, and lipid assembly contain the AW-box featured with [CnTnG](n)_7_[CG] (**Figure 6A**). To test whether BnWRI1 binds to the target DNA fragments containing the AW-box, the BnWRI1 protein was expressed in *E. coli* and purified for binding assays (**Figure 6C**). The DNA fragments of 250–300 bp that contained the AW-box [CnTnG](n)_7_[CG] in the promoter region of two representative genes known as *KASI* and *GPAT9* were amplified and used for EMSA. When BnWRI1 protein was incubated with the DNA fragment amplified from the *KASI* promoter containing the AW-box [CnTnG](n)_7_[CG], the target DNA was bound to BnWRI1, as indicated by the shifted bands at the top of gel when it was visualized under UV light (**Figure 6D**). However, when the DNA fragment with a mutant AW box [TnCnA](n)_7_[CG] was explored, the binding between the BnWRI1 and the DNA fragment was abolished, as shown by the observation that the DNA band position remaining unchanged in the gel (**Figures 6B,D**). A similar gel shift was observed when BnWRI1 protein was incubated with the DNA fragment containing an AW box [CnTnG](n)_7_[CG] from the promoter region of *GAPT9* (**Figure 6D**). The binding was diminished when the consensus was mutated to [CnTnG](n)_7_[TA] for the *GPAT9* promoter (**Figures 6B,D**). A similar result was found when the DNA probes were labeled with biotin (**Figure 6E**). These results suggest that BnWRI1 specifically binds to the promoter region containing the AW-box, which is conserved with *Arabidopsis* WRI1, and the AW-box is essential for the interaction between BnWRI1 and the target gene promoters.

### Overexpression of Bn*WRI1* Up-regulates the Transcript Level of Genes in Glycolysis, Fatty Acid Synthesis, Lipid Assembly, and Flowering

**FIGURE 7:**
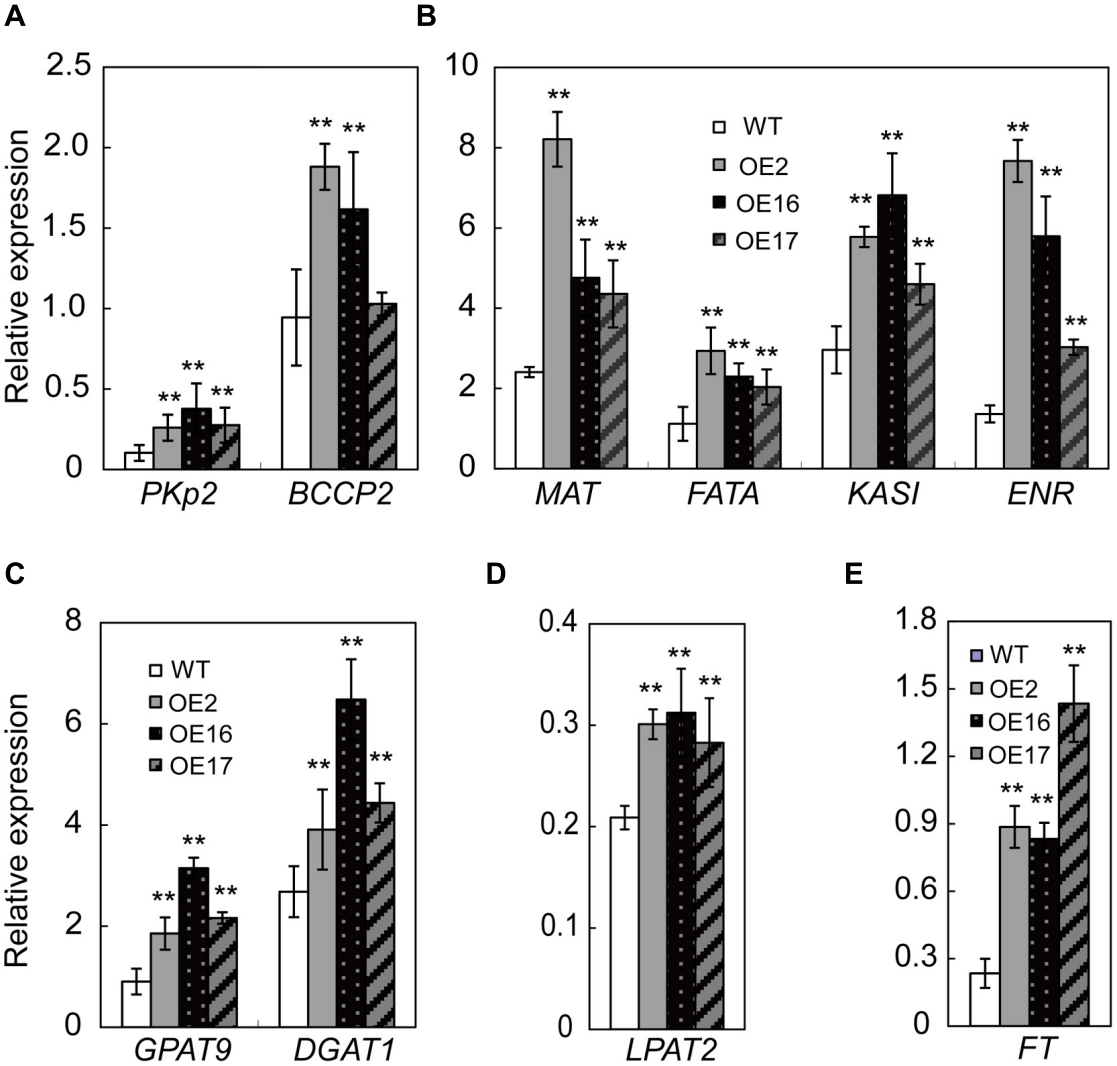
**Transcript levels of genes involved in lipid anabolism in Bn*WRI1-*OE and WT plants. (A,B)** The expression levels of genes involved in glycolysis and FA synthesis. **(C,D)** The expression levels of genes involved in lipid assembly. **(E)** The expression level of *FT* involved in flowering. Total RNA was extracted from the leaves of 7-weeks-old plants and the transcript levels of genes were analyzed by quantitative real-time PCR. *PKp2*, pyruvate kinase, homolog At5g52920; *BCCP2*, biotin carboxyl carrier protein, homolog At5g16390; *MAT*, malonyl-CoA:ACP malonyltransferase, homolog At2g30200; *KASI*, ketoacyl-ACP synthases, homolog At5g46290; *ENR1*, enoyl-ACP reductase, homolog At2g05990; *FATA*, acyl-ACP thioesterase, homolog At3g25110; *GPAT9*, glycerol-3-P acyltransferase, homolog At5g60620; *LPAT2*, lysophosphatidic acid acyltransferase, homolog At3g57650; and *DGAT1*, DAG acyltransferase, homolog At2g19450. The expression level was normalized to that of Bn*Actin*. Values are mean ± SD (*n* = 3); ^∗∗^*P* < 0.01.

To investigate whether Bn*WRI1* OE up-regulated the transcript level of genes involved in the lipid anabolism process, RNA was extracted from the leaves and analyzed by quantitative real-time PCR. These genes include *PKp2* in glycolysis, *BCCP2, MAT, KASI, ENR1*, and acyl-ACP thioesterase (*FATA*) in the FA biosynthetic process, and *GPAT9, LPAT2*, and *DGAT1* in lipid assembly. The transcript levels of the tested genes were all significantly higher in OE plants than in the WT. Despite the significant elevation of genes in multiple pathways by BnWRI1, the regulation of BnWRI1 in specific routes differs to variable extents. The transcript level of genes involved in glycolysis and FA synthesis including *PKp2, MAT, KASI, ENR1*, and *FATA* was up-regulated the most, and the expression levels in OE plants were more than twofold that of the WT (**Figures 7A,B**). The BnWRI1 enhanced expression of genes involved in FA synthesis was most prominent among the tested genes (**Figure 7B**). Moreover, RNA accumulation for the genes involved in lipid assembly was also strongly induced in OE plants. The mRNA level of *GPAT9* in OE plants accumulated more than twofold that of the WT (**Figure 7C**). The *LPAT2* and *DGAT1* transcript levels were also substantially higher in OE than in WT plants (**Figures 7C,D**). In addition, the FLOWERING LOCUS T (FT) is a key regulator in the control of flowering time in several plant species, and the *FT* expression level in OE plants was threefold higher than that of the WT (**Figure 7E**). The results suggest that BnWRI1 synchronously promotes multiple pathways in transcriptional regulation to enhance the plant reproductive process, seed development, and oil accumulation in *B. napus*.

### The Effect of BnWRI1 on the Membrane Lipid Composition

**FIGURE 8:**
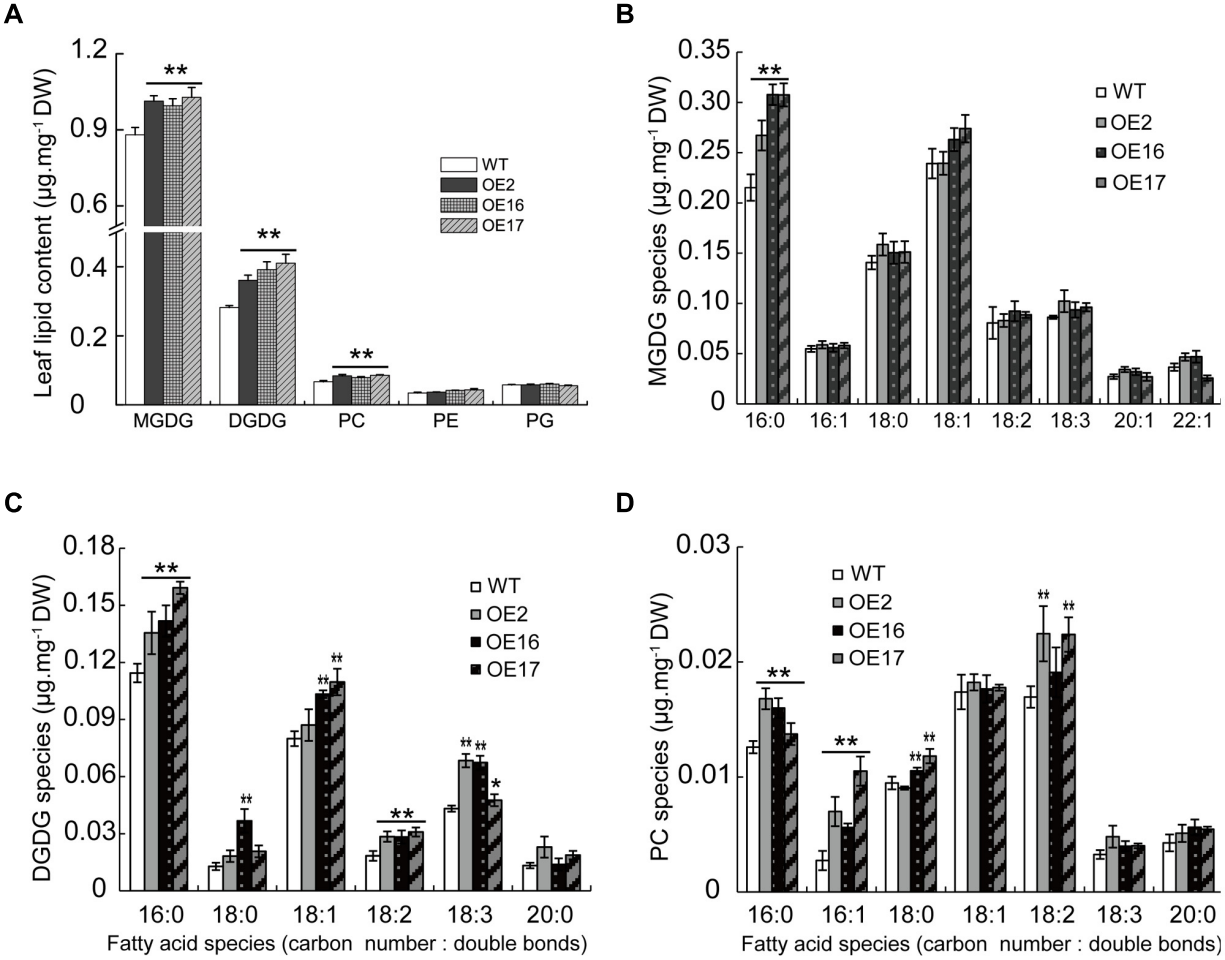
**Effect of Bn*WRI1-*OE on membrane lipid compositions in leaves. (A)** Lipid compositions in the leaves of Bn*WRI1*-OE and WT plants. Fatty acid species of MGDG **(B)**, DGDG **(C)**, and PC **(D)** in the leaves. Lipids were extracted from 5-month-old plants during the early flowering stage. The extracted lipids were separated by TLC plates and the spot corresponding to a specific lipid was quantitatively analyzed by GC measurement. Values are mean ± SE (*n* = 4); ^∗^*P* < 0.05, ^∗∗^*P* < 0.01.

Most studies have focused on the effect of WRI1 on oil accumulation, but the effect of WRI1 on the membrane lipid composition remains elusive. The OE of Bn*WRI1* in *B. napus* leads to the involvement of numerous genes in glycolysis, FA biosynthesis, and the lipid assembly process, indicating its significance in lipid anabolism. To investigate the effect of BnWRI1 on various lipid metabolisms further, phospholipids and galactolipids from leaves or siliques during flowering stages were analyzed. The phosphatidylethanol (PE) and phosphatidylglycerol (PG) in leaves remained comparable between OE and WT plants (**Figure 8A**). However, Bn*WRI1* OE resulted in a significant increase in MGDG, DGDG, and PC in leaves relative to their levels in WT plants (**Figure 8A**). The increased DGDG resulted from the increase in 16:0, 18:1, 18:2, and 18:3 FA species, and the elevated MGDG primarily came from an increased 16:0 FA, whereas the enhanced PC level was mostly contributed by increased 16:0, 16:1, and 18:2 FAs (**Figures 8B–D**).

**FIGURE 9:**
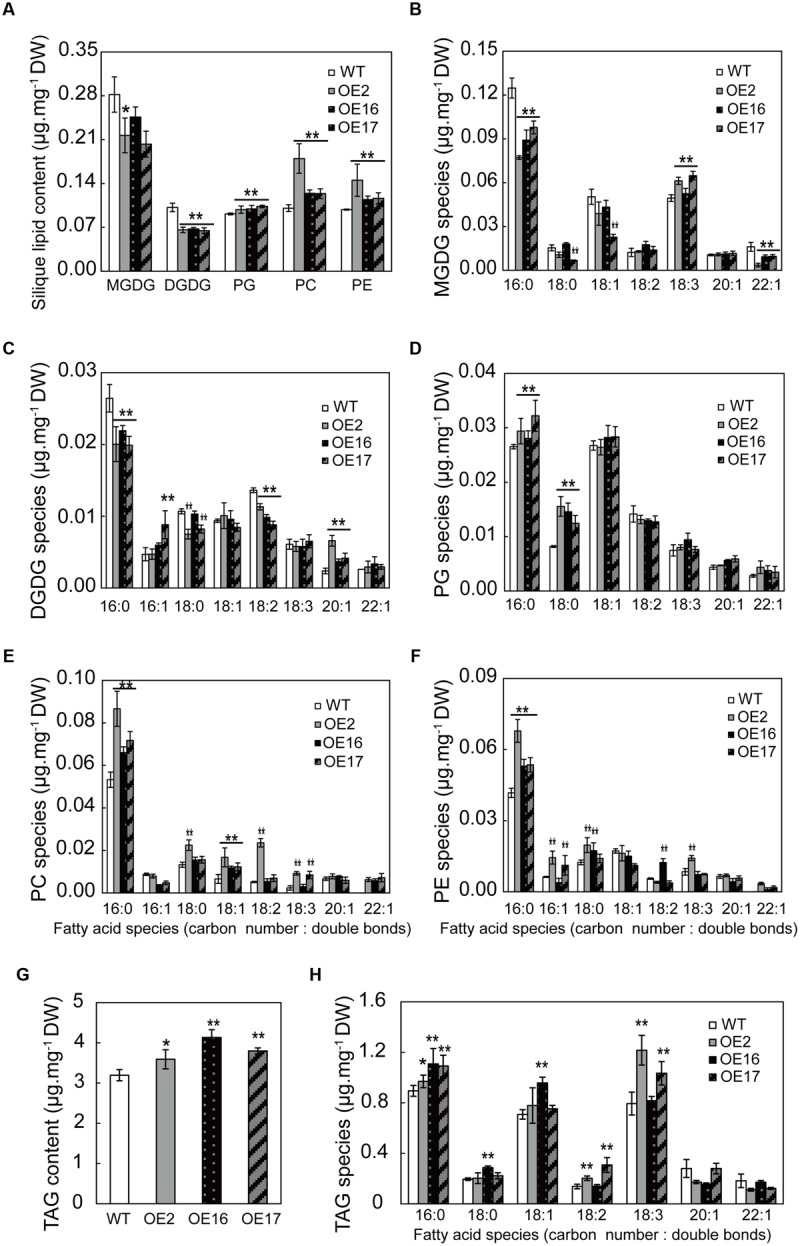
**Effect of Bn*WRI1*-OE on membrane lipids and TAG levels in siliques. (A)** Lipid composition in the siliques of Bn*WRI1*-OE and WT plants. Lipids were extracted from the siliques at 20 days after anthesis (DAA). The extracted lipids were separated by TLC and the spot corresponding to a specific lipid was quantitatively analyzed by GC. **(B–F)** FA species of MGDG, DGDG, PG, PC, and PE in the siliques of 20 DAA. Oil (TAG) content **(G)** and FA species of TAG **(H)** in the siliques of 20 DAA. Values are mean ± SE (*n* = 4); ^∗^*P* < 0.05, ^∗∗^*P* < 0.01.

Lipid profiling from developing siliques revealed that the lipid content and composition of siliques were significantly different from those of leaves. The contents of galactolipids such as DGDG and MGDG in siliques, the predominant components of chloroplast membranes, were much lower than the contents in leaves (**Figures 8** and **9**), suggesting that photosynthesis predominantly occurs in leaves rather than siliques during the flowering and early seed development stages. Bn*WRI1* OE resulted in increased phospholipids and a corresponding reduction in galactolipids (**Figure 9A**). OE siliques contain less DGDG with decreased 16:0, 18:2 FAs and increased 20:1 FA in OE siliques (**Figure 9C**). MGDG was also reduced with decreased 16:0, 22:1 FA and increased 18:3 FA in OE siliques, and the magnitude was smaller than that of DGDG (**Figures 9A–C**). By comparison, phospholipids PC, PE, and PG in OE siliques were higher than the levels in the WT (**Figure 9A**). The increased PC and PE in OE siliques were contributed mostly by increased 16:0 FA, whereas elevated PG resulted from increased 16:0 and 18:0 FAs in OE siliques. (**Figures 9D–F**). The TAG in OE siliques was significantly higher than that of the WT, and increased TAG comes primarily from elevated 16:0 FA. The 18:2 and 18:3 FA contents in both OE2 and OE17 were also higher than those in WT siliques (**Figures 9G,H**).

## Discussion

Fatty acids are major, basic components of lipid assembly, and their destinations include membrane lipids, storage lipids, lipid messengers, and other derivatives. The FA synthesis enzyme complex consists of multiple subunits including malonyl-CoA: ACP transferase (MAT), acyl-ACP transferase (AT), β-ketoacyl-ACP synthase (KAS), β-ketoacyl-ACP reductase (KAR), hydroxyacyl-ACP dehydrogenase (HAD), and enoyl-ACP reductase (ENR) as encoded by individual genes in plants (Slabas and Fawcett, 1992; Ohlrogge and Browse, 1995; Ohlrogge and Jaworski, 1997). WRI1 is a central regulator that modulates numerous genes and multiple steps in oil synthesis simultaneously, and it is unique to plants (Cernac and Benning, 2004; Shen et al., 2010). However, most studies are focused on WRI1 involvement in oil accumulation in *Arabidopsis*. The effect of WRI1 on lipid synthesis and its consequence on plant development were unknown, especially in crop plants. This study unraveled the novel roles of BnWRI1 in flowering time control and lipid homeostasis in relation to oil accumulation, membrane phospholipids, galactolipids, and sugars. Enhanced lipid anabolism and accelerated flowering from Bn*WRI1* OE did not result in the inhibition of protein synthesis and total sugar accumulation. Unlike the results found in *Arabidopsis* (Cernac and Benning, 2004), Bn*WRI1* OE in *B. napu*s plants did not cause visible undesirable growth or development traits. These results suggest that the roles of BnWRI1 in *B. napus* is conserved and yet distinguishable from that of other species such as *Arabidopsis* and maize (Focks and Benning, 1998; Baud et al., 2007; Pouvreau et al., 2011; To et al., 2012).

A previous study showed that the AP2-type transcription factors play important roles in regulating meristem growth and organ development. AP2-type transcription factors are also involved in ovule development, floral organ growth, and seed size (Jofuku et al., 1994; Elliott et al., 1996; Okamuro et al., 1997; Mizukami and Fischer, 2000; Ohto et al., 2005). WRI1 belongs to the AP2-type transcription factor family, but its role in the flowering time had not been reported. Our result showed that Bn*WRI1*-OE plants flowered 4–6 days earlier than the WT, suggesting that BnWRI1 plays a role in flowering time control. WRI1 from *Arabidopsis* or maize is not involved in flowering time control (Cernac and Benning, 2004; Pouvreau et al., 2011). Early flowering is an important trait because seeds can be matured within a given time to make field space available for the next crop’s growth. In addition to its role in oil accumulation, a mild earlier flowering by Bn*WRI1*-OE did not lead to a visible inhibition in vegetative growth and biomass, which provides a possibility for oil crop breeding. Gene expression profiling showed that Bn*WRI1*-OE plants enhanced *FT* expression, suggesting that BnWRI1 modulates flowering in a transcriptional manner. However, the promoter region of *FT* does not contain a typical AW-box, suggesting that other *cis* elements in the *FT* promoter are recognized and regulated by BnWRI1. Alternatively, the early flowering may have resulted from PC elevation as shown by the increased PC level in OE plants. Recent studies showed that PC plays a positive role in *Arabidopsis* flowering (Nakamura et al., 2014; Wang et al., 2015).

An oil component in the form of TAG is primarily derived from sugars through photosynthesis in plants. In oil seed plants such as *B. napus* and *Arabidopsis*, starch was accumulated at the early phase of seed development and converted to TAG and proteins at the later phase of seed maturation (Baud et al., 2002; Ruuska et al., 2002; Hills, 2004). Our study showed that Bn*WRI1* OE in *B. napus* resulted in significant oil accumulation in both seeds and leaves, which was accompanied by up-regulated genes involved in glycolysis, FA biosynthesis, and lipid assembly, whereas the AtWRI1 in *Arabidopsis* was not involved in lipid assembly (Cernac and Benning, 2004; Baud et al., 2007; To et al., 2012). The seed-specific OE of Bn*WRI1* in *B. napus* or in *Arabidopsis* resulted in enhanced seed oil content and seed size (Liu et al., 2010; Wu et al., 2014). The OE of *Arabidopsis WRI1* in *Camelinasativa* also led to increased oil content and seed size (An and Suh, 2015). AtWRI1 binds to the AW-box [CnTnG](n)_7_[CG] located at the proximal upstream regions of genes involved in glycolysis and FA synthesis (Maeo et al., 2009). BnWRI1 contains two AP2 domains involved in DNA binding. Our results showed that BnWRI1 was localized to the nucleus. An EMSA assay showed that BnWRI1 was capable of binding to the AW-box [CnTnG](n)_7_[CG] at the proximal promoter region of genes involved in FA synthesis and lipid assembly, and the AW-box is essential for binding because the nucleotide substitutive mutant abolished the binding. Moreover, lipid assembly is also important for oil synthesis as demonstrated by overexpressing the genes encoding yeast glycerol-3-phosphate dehydrogenase and yeast LPAT in *B. napus* leading to a modest increase in oil content (Zou et al., 1997; Vigeolas et al., 2007). Likewise, an elevated *DGAT* transcript level in soybeans and maize resulted in enhanced seed oil content (Zheng et al., 2008). The current study showed that BnWRI1 also binds to the promoter region of *GPAT9* that is responsible for lipid assembly, which is not found in *Arabidopsis* and other plant species (Baud et al., 2007; To et al., 2012). These observations suggest that the transcriptional regulation of BnWRI1 and its effect on the lipid anabolic process is more comprehensive in *B. napus* than in other plant species.

As fossil energy becomes limited, it is attractive to enhance oil accumulation in vegetative tissues to supply renewable biofuel and feed stock for industry and animal foods. The current results showed that Bn*WRI1*-OE in *B. napus* led to increased oil contents in the leaves without growth retardation, suggesting a potential application in crop plant breeding. However, the increased amount of leaf oil content is moderate at 1.4% of the dry weight, which is similar to that of tobacco leaves that transiently expressed *WRI1* from other plant species (Grimberg et al., 2015). A transcriptional analysis showed that WRI1 up-regulated genes in both FA synthesis and degradation, indicating a futile cycle of FA metabolism, which may be responsible for the limitation in leaf oil accumulation (Grimberg et al., 2015). The results suggest that achieving high oil contents in the leaves through single gene manipulation could be challenging. A recent study showed that the co-expression of three genes, *WRI1, DGAT* and o*leosin*, led to 15% TAG dry weight in tobacco leaves (Vanhercke et al., 2014).

Carbon allocation by WRI1 occurred in *Arabidopsis* and cotton plants. The constitutive expression of At*WRI1* in *Arabidopsis* led to a significant increase in the oil content, which was accompanied by defective seed germination and plant growth (Cernac and Benning, 2004). Suppressing WRI1 in cotton led to increased fiber length and reduced seed oil content (Qu et al., 2012). Our results showed that the total sugars and proteins remained constant between OE and WT, but OE plants displayed reduced carbohydrate storage with a corresponding increase in soluble sugars in the leaves and seeds. In addition to oil accumulation, Bn*WRI1* OE also increased phospholipids and galactolipids significantly in leaves. Higher levels in Bn*WRI1*-OE leaves of the galactolipids MGDG and DGDG, the important components of chloroplast thylakoid membranes that are essential for photosynthesis, may be responsible for enhanced source capacity. A recent study showed that the seed-specific expression of Bn*WRI1* promoted the expression of the photosynthesis gene (Wu et al., 2014). The results suggest that the enhanced lipid anabolic process in leaves by BnWRI1 is not a result of a reduction in other organic components but a consequence of enhanced source/sink strength by enhanced galactolipids and carbon flux to lipid accumulation. This finding may explain, at least in part, how Bn*WRI1*-OE enhances oil accumulation and flowering without growth inhibition in *B. napus*. However, the lipid metabolism regulated by BnWRI1 in siliques is quite different from that of leaves. A lipid analysis of the siliques revealed reduced galactolipids MGDG and DGDG, and corresponding increases in the phospholipids PC, PE, PG, and storage lipid TAG in OE siliques during flowering and early seed developing stages. These results indicate that enhanced PC and PE may facilitate lipid mobilization to oil accumulation in seeds because TAG can be directly derived from PC by PDAT activation (Dahlqvist et al., 2000; Fan et al., 2013, 2014). Our data support the facts that BnWRI1 regulates homeostasis among sugars, membrane lipids, and storage lipids, and its consequences for enhancing reproduction, seed development and oil accumulation. Our results shed light on the roles of BnWRI1 in lipid regulatory networks and its application in oil crop plant breeding.

**FIGURE 10:**
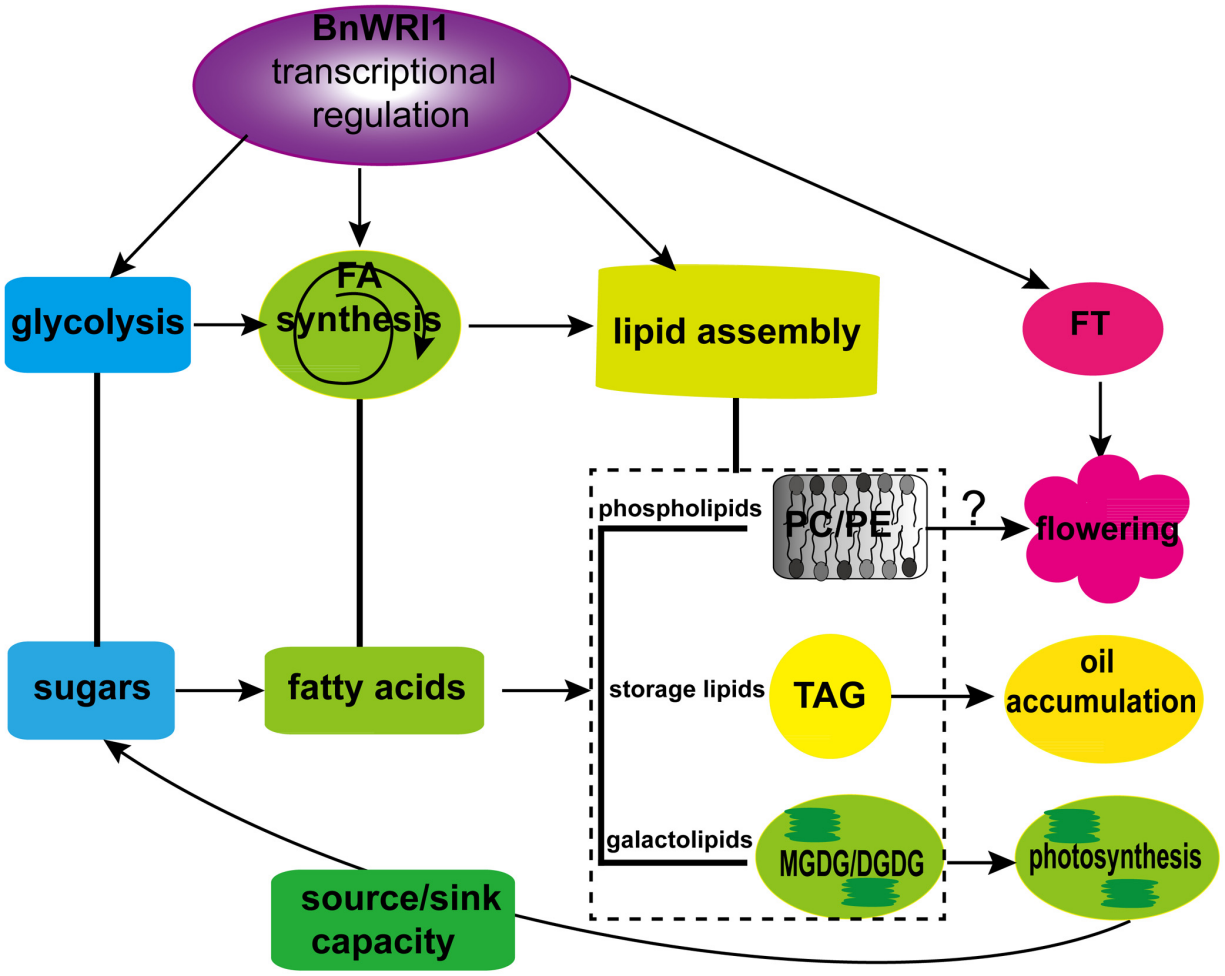
**A proposed model for BnWRI1 in regulating lipid anabolism and flowering in *B. napus*.** BnWRI1 up-regulates transcript levels of genes involved in glycolysis, FA synthesis, and lipid assembly to enhance accumulation of oil (TAG), galactolipids, and phospholipids. The increased MGDG and DGDG by BnWRI1 promote source/sink capacity, thus BnWRI1 plays a positive role in homeostasis among sugars, TAG, and membrane lipids in *B. napus*. Moreover, BnWRI1 also enhances *FT* expression, and PC level to accelerate flowering.

In summary, the results from this study identified several novel roles of BnWRI1 in *B. napus* that have not been characterized before. First, the OE of Bn*WRI1* led to accelerated plant flowering 4–6 days earlier without reduced vegetative growth, which is a good agronomic trait to open up field space for the next crop’s planting. Second, BnWRI1 enhanced oil accumulation in both seeds and leaves without visible side effects on growth. BnWRI1 decreased storage carbohydrates and increased soluble sugars to facilitate carbon flux to lipid anabolism. Third, BnWRI1 is localized in the nucleus and binds to the AW-box at the proximal promoter region of genes involved in FA synthesis as well as in the lipid assembly process that is not found in *Arabidopsis*. The OE of Bn*WRI1* led to the up-regulated transcription of genes in the lipid anabolic pathway, and of *FT* in flowering control. Finally, BnWRI1 not only enhances oil accumulation, but it also affects membrane lipid metabolism and turnover in both leaves and developing siliques. Bn*WRI1* OE caused increased galactolipid MGDG, DGDG, and phospholipid PC in leaves, but it led to reduced DGDG and MGDG and increased PC, PE, and TAG in siliques during the early seed development stage. Enhanced galactolipid synthesis by BnWRI1 is beneficial for photosynthesis to enhance the source/sink capacity, and thus facilitates the sugar flux to oil accumulation without the expense of sugar and protein in both leaves and seeds. Therefore, BnWRI1 plays a positive role in homeostasis among sugars, membrane lipids, and oil accumulation by modulating the coordination of multiple metabolism pathways, thus enhancing flowering and oil accumulation without growth inhibition. Based on the results from this study, we proposed a work model for BnWRI1 in lipid anabolic process, plant growth and development (**Figure 10**). The early flowering led by BnWRI1 may be caused by its transcriptional regulation through binding to the promoter region of *FT* or other flowering-related genes. It would be interesting to identify new targets of BnWRI1 that are involved in flowering control in future studies. In addition, Bn*WRI1* OE led to an elevated PC level in both leaves and siliques. PC functions not only as an essential component of the membrane structure, but it also plays important roles in flowering regulation and providing acyl chains for TAG accumulation (Dahlqvist et al., 2000; Zhang et al., 2009; Nakamura et al., 2014; Wang et al., 2015). It would be interesting to explore how PC synthesis is regulated by BnWRI1 in future work.

## Author Contributions

YH, QL, and JS designed the study. QL and JS performed most of the experiments; ST helped cloning and transformation; QS performed EMSA; TW analyzed some of metabolites; WC helped isolation and identification of transgenic plants.

## Conflict of Interest Statement

The authors declare that the research was conducted in the absence of any commercial or financial relationships that could be construed as a potential conflict of interest.
