# Corrigendum: Wrinkled1 Accelerates Flowering and Regulates Lipid Homeostasis between Oil Accumulation and Membrane Lipid Anabolism in *Brassica napus*

**DOI:** 10.3389/fpls.2015.01270

**Published:** 2016-01-19

**Authors:** Qing Li, Jianhua Shao, Shaohua Tang, Qingwen Shen, Tiehu Wang, Wenling Chen, Yueyun Hong

**Affiliations:** National Key Laboratory of Crop Genetic Improvement, College of Life Science and Technology, Huazhong Agricultural UniversityWuhan, China

**Keywords:** Wrinkled1 (WRI1), oil accumulation, flowering, lipid homeostasis, transcriptional regulation, *Brassica napus*

Reason for Corrigendum:

There was a mistake in the unit of the y-axis legend of Figure 4A as published. The correct unit should be “μg.mg^−1^ DW” as in the new version of Figure 4A, appears below. The authors apologize for the mistake. This error does not change the scientific conclusions of the article in any way.

**Figure 4A:**
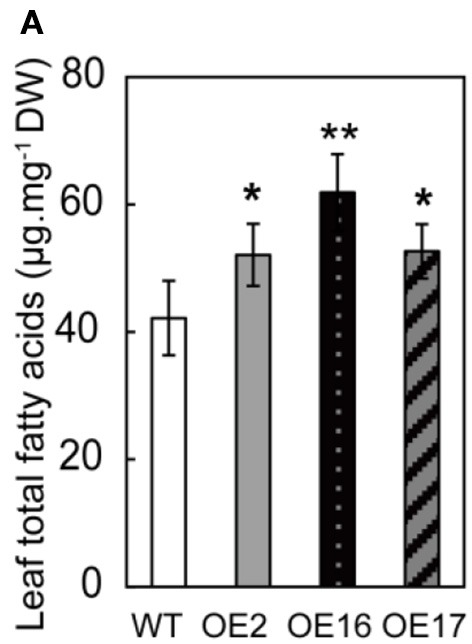


## Conflict of interest statement

The authors declare that the research was conducted in the absence of any commercial or financial relationships that could be construed as a potential conflict of interest.

