# Supplementary material for: Wrinkled1 Accelerates Flowering and Regulates Lipid Homeostasis between Oil Accumulation and Membrane Lipid Anabolism in Brassica napus
Source: Front Plant Sci. 2015 Nov 19;6:1015. doi: 10.3389/fpls.2015.01015 (PMC4652056; doi:10.3389/fpls.2015.01015)
Supplement: Supplementary file 1 [file Table_1.DOC]

**Supplemental Table 1. Primers used for constructs and binding assay**

| Purposes | Primer name | Primer sequence |
| --- | --- | --- |
| Bn*WRI1* OE | Bn*WRI1*-F | 5’-GGATCCATGAAGAGACCCTTAACCAC-3’ |
|  | Bn*WRI1*-R | 5’-GAGCTCTCAGACAGAATAGTTCCAAGAA-3’ |
| Bn*WRI1* OE  Identification | pBI121-35s  Bn*WRI1*-R | 5’-CACTATCCTTCGCAAGACC-3’  5’-GAGCTCTCAGACAGAATAGTTCCAAGAA-3’ |
| BnWRI1-His | Bn*WRI1*-F28 | 5’-GAATTCATGGCACCTCCAAAAGCCCTTG-3’ |
|  | Bn*WRI1*-R28 | 5’-CTCGAGTTCATTGTACTTGGTAATTGTCCTCCG-3’ |
| BnWRI1-GFP | *WRI1*-F(GFP) | 5’-GAGCTC ATGAAGAGACCCTTAACCAC-3’ |
|  | *WRI1*-R(GFP) | 5’-GGATCC GACAGAATAGTTCCAAGAAAC-3’ |
| Actin control | Bn*actin*-F  Bn*actin*-R | 5’-AGCTGGAGACGGCTAAGAG-3’  5’-GTTGGAAAGTGCTGAGGGA-3’ |
| Binding DNA sequence | *KASI*-F | 5’-GAATTCTGTTGAGTTACGAATTGGAG-3’ |
|  | *KASI*-R | 5’-GAGCTCATTGAGAGAGGTATTGAGAG-3’ |
|  | *GPAT9*-F | 5’-GAATTCACATAATATGTCCAAGATCATT-3’ |
|  | *GPAT9*-R | 5’-GAGCTCCTATTATACTTATACCACAT-3’ |
|  | *KASI*-Fm | 5’-AATAATCGAGTTCAAGCTAAAACTGAAGACAA-3’ |
|  | *KASI*-Rm | 5’-TATAAAAAAAACAGCGAATTATTTGGGAAAAT-3’ |
|  | *GPAT9*-Fm | 5’-ATTAACTTTCTTTGGAGAGTTACTGT-3’ |
|  | *GPAT9*-Rm | 5’-AATATTTCTTTCAATATCTAGATTCT-3’ |

**Supplemental Table 2. Primers used for quantitative real time PCR**

| Primer name | Primer sequence |
| --- | --- |
| Bn*actin*-F | 5’-TGTTCCCTGGAATTGCTGACCGTA-3’ |
| Bn*actin*-R | 5’-TGCGACCACCTTGATCTTCATGCT-3’ |
| Bn*WRI1*-RT-F | 5’-CCAGGCATCACCATAACGGAAGAT-3’ |
| Bn*WRI1*-RT-R | 5’-CTGCAGCTTCCTCCTGCGTATTAT-3’ |
| Bn*PKp2*-RT-F | 5’-TGCCAGAAGGGCGATAGTGACTTA-3’ |
| Bn*PKp2*-RT-R  Bn*ENR1*-RT-F  Bn*ENR1*-RT-R  Bn*FATA*-RT-F  Bn*FATA*-RT-R  Bn*BCCP2*-RT-F  Bn*BCCP2*-RT-R  Bn*DGAT1*-RT-F  Bn*DGAT1*-RT-R | 5’-ATGCCACCTAATCTTGGTCAAGCC-3’  5’-CCCAGGAGGTGCTTCTATATCT-3’  5’-CACCCGTGTATCACTCTCTAGT-3’  5’-CAGGAGATCAATCGTGGAAGAA-3’  5’-ACCAACACTGGAGAAGCATATAA-3’  5’-CGATGAGGGAGGTGGTTAAAG-3’  5’-TGGTTGGGAGGGTGATA-3’  5’-GCAGTTCCTTGTCGTCTCTT-3’  5’-CATGGAGCCAAACCTTTCTTG-3’ |
| Bn*MAT*-RT-F | 5’-TTGGCGGAAACGGAGATCAGAAGT-3’ |
| Bn*MAT*-RT-R | 5’-AGATGTCACCTGGCGTGCAAGTAT-3’ |
| Bn*KASI*-RT-F | 5’-TGGTGGGACTGAGGCTGCTATTAT-3’ |
| Bn*KASI*-RT-R | 5’-GCCGTTTGAGGGTCATCATTTCTCTG-3’ |
| Bn*GPAT9*-RT-F | 5’-TGTTGACGCCTTCTGGAATAG-3’ |
| Bn*GPAT9*-RT-R | 5’-TCCAAGTACCACACTTCACATAC-3’ |
| Bn*LPAT2*-RT-F | 5’-GTGGCTAAGGATGCTCTGTT-3’ |
| Bn*LPAT2*-RT-R | 5’-CCACCGCAAGGGACTTTAT-3’ |
| Bn*FT*-RT-F | 5’-CAGTTAAGCAGTGATGAGCAAAG-3’ |
| Bn*FT*-RT-R | 5’-GGGAAGATGGTGTGGAGTAAAG-3’ |
